# Emerging roles of circular RNAs in osteoporosis

**DOI:** 10.1111/jcmm.16906

**Published:** 2021-09-06

**Authors:** Weichun Chen, Baozhong Zhang, Xiao Chang

**Affiliations:** ^1^ Department of Orthopaedic Surgery Peking Union Medical College Hospital Chinese Academy of Medical Sciences and Peking Union Medical College Beijing China

**Keywords:** ceRNA, circRNAs, microRNA, osteoporosis

## Abstract

Osteoporosis is one bone disease characterized with skeletal impairment, bone strength reduced and fracture risk enhanced. The regulation processes of bone metabolism are associated with several factors such as mechanical stimulation, epigenetic regulation and hormones. However, the mechanism of osteoporosis remains unsatisfactory. Increasing high‐throughput RNA sequencing and circular RNAs (circRNAs) microarray studies indicated that circRNAs are differentially expressed in osteoporosis. Growing functional studies further pinpointed specific deregulated expressed circRNAs (e.g., circ_28313, circ_0016624, circ_0006393, circ_0076906 and circ_0048211) for their functions involved in bone metabolism, including bone marrow stromal cells (BMSCs) differentiation, proliferation and apoptosis. Moreover, CircRNAs (circ_0002060, Circ_0001275 and Circ_0001445) may be acted as diagnostic biomarkers for osteoporosis. This review discussed recent progresses in the circRNAs expression profiling analyses and their potential functions in regulating BMSCs differentiation, proliferation and apoptosis.

## 1 INTRODUCTION

Osteoporosis is one bone disease characterized with skeletal impairment, bone strength reduced and fracture risk enhanced.^1^, ^2^, ^3^, ^4^ It is accompanied or asymptomatic by mild or serious symptoms, which is one cause of pathological fracture and also the high‐risk factor influencing human health.^5^, ^6^, ^7^ The incidence of osteoporosis is 70% in people over 80 years and 15% in people over 50 years old.^8^, ^9^ The osteoporosis patient's number exceeds 200 million worldwide at present, while the number may increase to 300 million as ageing population by 2023.^10^ The modulation processes of bone metabolism are associated with several factors such as mechanical stimulation, epigenetic regulation and hormones.^11^, ^12^, ^13^, ^14^, ^15^ The major therapies of osteoporosis are drug therapy and surgery, while the curative effect remains unsatisfactory.^16^, ^17^, ^18^ Thus, it is crucial to find new biomarkers for the indention and therapy of osteoporosis.

Circular RNAs (circRNAs) are formed by reverse splicing of splice accepter at 5′ end splice donor at the 3′ end in the pre‐mRNA.^19^, ^20^, ^21^, ^22^ circRNAs are one group of noncoding RNA and temporal, disease specific and spatial are expressed in several tissues and cells and can be acted as therapeutic targets and biomarkers.^23^, ^24^, ^25^, ^26^ Although circRNAs are mostly located in cytoplasm, some circRNAs containing introns are originated from nucleus.^27^ CircRNAs have several functions including regulating gene transcription, regulating translation, modulating alternative splicing, functioning as miRNA sponges and interacting with RBPs (RNA‐binding proteins).^28^, ^29^, ^30^, ^31^, ^32^ Growing evidence suggested that circRNAs play crucial roles in cell functions such as cell metabolism, differentiation, proliferation, apoptosis and invasion.^19^, ^33^, ^34^, ^35^, ^36^ Increasing studies showed that circRNAs involved in the development of many diseases including cancers, neurological diseases, congenital diseases, intervertebral disc degeneration and endocrine diseases.^37^, ^38^, ^39^, ^40^, ^41^, ^42^, ^43^ Recently, studies indicated that circRNAs also play important roles in osteoporosis.^44^

In the manuscript, we reviewed expression profiling of circRNA studies in osteoporosis to offer the datasets for choosing specific osteoporosis‐associated circRNAs for next studies in the future. The potential diagnostic and therapeutic roles of circRNAs in the clinical application for osteoporosis are also discussed.

## 2 CIRCRNA EXPRESSION PROFILING AND INTEGRATIVE ANALYSIS IN SCI

Huang et al.^45^ used circRNA chip analysis to study circRNA expression profiling in the plasma or serum from 40 osteoporosis patients and 40 control adults. A total of 237 circRNAs were found to be differently expressed and 95 circRNAs were downregulated and 162 circRNAs were upregulated. Moreover, they performed qRT‐PCR analysis to prove that four circRNAs such as circ_0081047, circ_0068459, circ_0006873 and circ_0002060 were upregulated and circ_0017736 and circ_0062582 were downregulated in osteoporosis patients compared to control adults.

Zhao et al.^46^ performed circRNA microarray to analysis circRNA expression profiling in PBMCs (peripheral blood mononuclear cells) from three postmenopausal osteoporosis patients and three controls. There were 381 circRNAs were differently expressed in postmenopausal osteoporosis compared to control. Among these, 203 circRNAs were overexpressed and 178 circRNAs were downregulated. They performed qRT‐PCR assay to confirm that circ_0028882, circ_0001275, circ_0006766, circ_0007788 and circ_0003391 was upregulated and circ_0006801 was downregulated.

Jin et al.^47^ used RNA sequencing to study circRNA expression profiling in three cases with postmenopausal osteoporosis and three healthy controls. A total of 260 circRNAs were differentially regulated in postmenopausal osteoporosis group. Among these, 154 circRNAs were downregulated and 106 circRNAs were overexpressed in osteoporosis group. The top five downregulated circRNAs were circ_0021739, circ _0011269, circ_0019693, circ_0005245 and circ_0010349, and the top five upregulated circRNAs were circ_0010452, circ_0022348, circ_0015566, circ_0003323 and circ_0013121.

Chen et al.^48^ performed microarray profiling analyses to detect the cirRNA expression pattern in the bone marrow monocyte/macrophage‐derived osteoclasts and undifferentiated BMM cells. A total of 6259 circRNAs were decreased, and 5449 circRNAs were overexpressed after BMM cells induction. Moreover, a total of 81 circRNAs were remarkably different by hierarchical clustering and 52 circRNAs were downregulated and 29 circRNAs were upregulated. They confirmed that the expression of circ_012460, circ_8313, circ_28312, circ_28309, circ_40206 and circ_28236 were overexpressed in induction group.

Yu et al.^49^ used RNA sequencing and bioinformatics assay to screen for circRNAs expression profiling in six postmenopausal osteoporosis patients and healthy controls. A total of 387 circRNAs were found to be differentially expressed in the osteoporosis compared to control including 176 decreased circRNAs and 211 overexpressed circRNAs. Moreover, they showed that circ_0057340 and circ_0134944 were overexpressed and circ_0005692, circ_0088422 and circ_0076906 were decreased in osteoporosis group by qRT‐PCR analysis.

Lin et al.^50^ have studied the circRNAs expression profiling in the osteoclast differentiation without and with alendronate treatment. There were 1394 circRNAs were upregulated and 214 circRNAs were downregulated in the osteoclast (OC) precursors (OPCSs) groups compared to OC group. GO assay showed that differentially expressed circRNAs were distributed into three groups: molecular function, biological process and cellular component. In addition, a total of 110 circRNAs were deregulated expressed among OC +alendronate, OC and OPCS groups and 15 circRNAs were downregulated and 95 circRNAs were overexpressed after alendronate treatment. The expression of circ_0000284, circ_0000638, circ_0000994, circ_0001776, circ_0002922, circ_0007710 and circ_0113954 was upregulated in the OC group compared to OPCS group.

Wang et al.^51^ used RNA sequencing to analyse aberrantly expressed circRNAs in BMSCs from ovariectomy mice and controls. There are 45 circRNAs were found to be differentially expressed and 21 circRNAs were decreased and 24 circRNAs were overexpressed in the ovariectomy mice compared to controls. Moreover, they confirmed that circ‐0020 expression was overexpressed and circ‐3832 level was downregulated in ovariectomy mice compared to controls.

Xu et al.^52^ used circRNAs microarray to study circRNAs expression profiling in the plasma or serum from osteoporosis and controls. A total of 69 circRNAs were differentially expressed in osteoporosis group compared to control groups, and among these, 35 circRNAs were decreased and 34 circRNAs were overexpressed. They performed qRT‐PCR assay to prove that circ_0019693, circ_0011269, circ_0028958, circ_0005245, circ_0006487 and circ_0010452 expression was consistent with the microarray data.

Shen et al.^53^ used circRNA microarray assay to study circRNAs expression profiling in bone tissues from osteoporosis group and no‐osteoporosis group. A total of 4972 circRNAs were differentially expressed in osteoporosis group compared to no‐osteoporosis group, and among these, 2645 circRNAs were overexpressed and 2327 circRNAs were decreased.

Yao et al.^54^ performed RNA sequencing to identify circRNAs expression profiling in peripheral blood from three patients from senile osteoporotic vertebral compression fracture (OVCF) and three healthy controls. They discovered that there are 884 circRNAs were differentially expressed and 330 circRNAs were downregulated and 554 circRNAs were overexpressed in OVCF groups compared to healthy controls.

Liu et al.^55^ performed RNA sequencing to investigate circRNAs expression profiling in five postmenopausal osteoporosis patients with five normal controls. There were 250 circRNAs were deregulated in osteoporosis compared to normal controls. Among these, 186 circRNAs were upregulated and 64 circRNAs were downregulated.

Wang et al.^56^ explored circRNAs expression profiling in the BMSCs after treated with melatonin or not by using RNA sequencing. They found that there were 209 circRNAs were differentially expressed in human BMSCs after treated with melatonin. Among these, there were 36 circRNAs were downregulated and 173 circRNAs were upregulated. They confirmed that the expression of circ_0002770, circ_0073244, circ_0003126, circ_0002867, circ_0008210, circ_0037026, circ_0005015, circ_0003865 and circ_0006935 was downregulated.

Zhi et al.^57^ performed the circRNA microarray to detect the differentially expressed circRNAs in serum of three controls and three osteoporosis patients. There were 589 circRNAs were differentially expressed in the serum in osteoporosis patients compared to controls. Among these, 213 circRNAs were decreased and 376 circRNAs were overexpressed in the osteoporosis patients.

**FIGURE 1:**
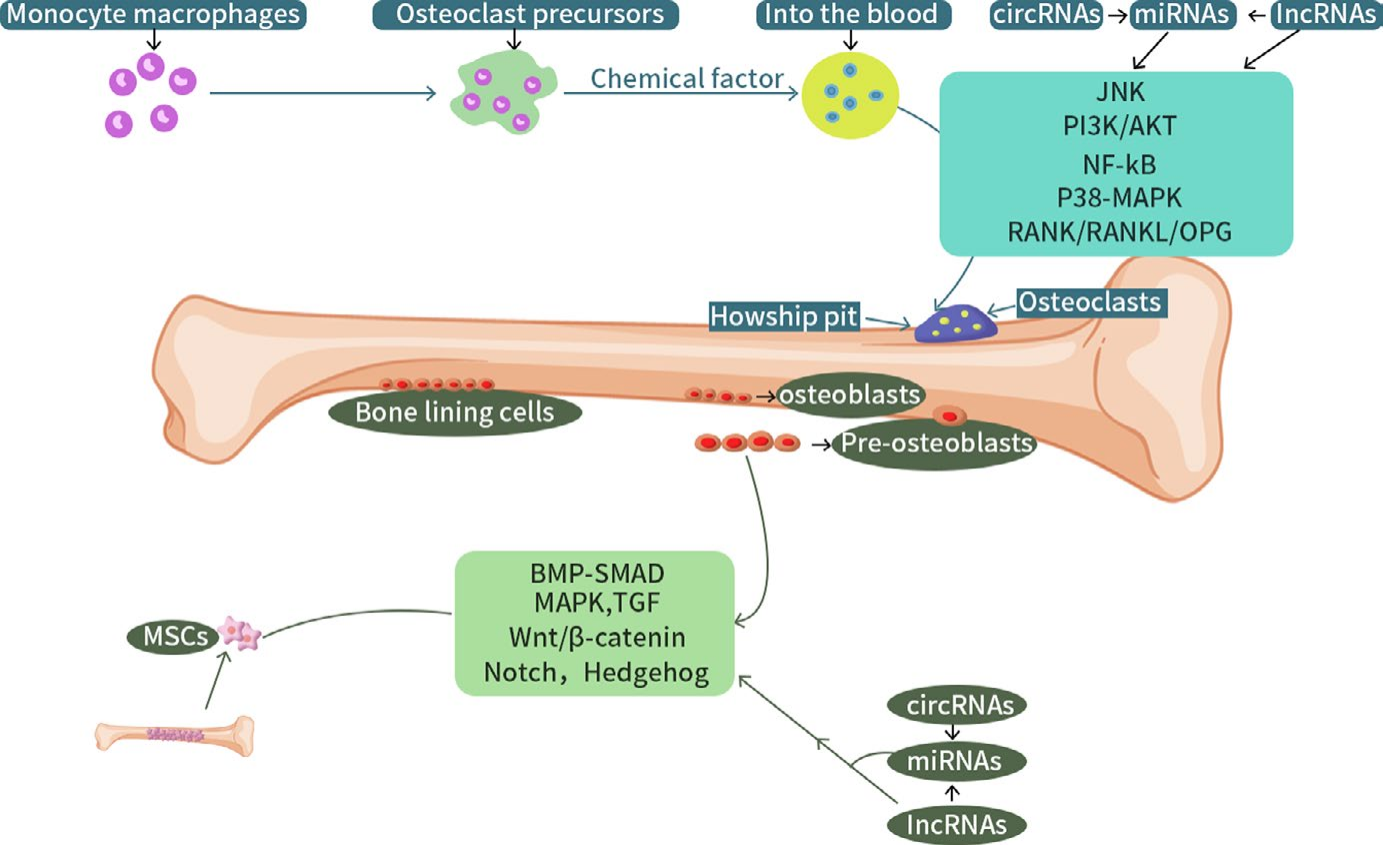
miRNA, lncRNA and circRNA affect osteoblasts and osteoclasts and participate in the development of osteoporosis

**FIGURE 2:**
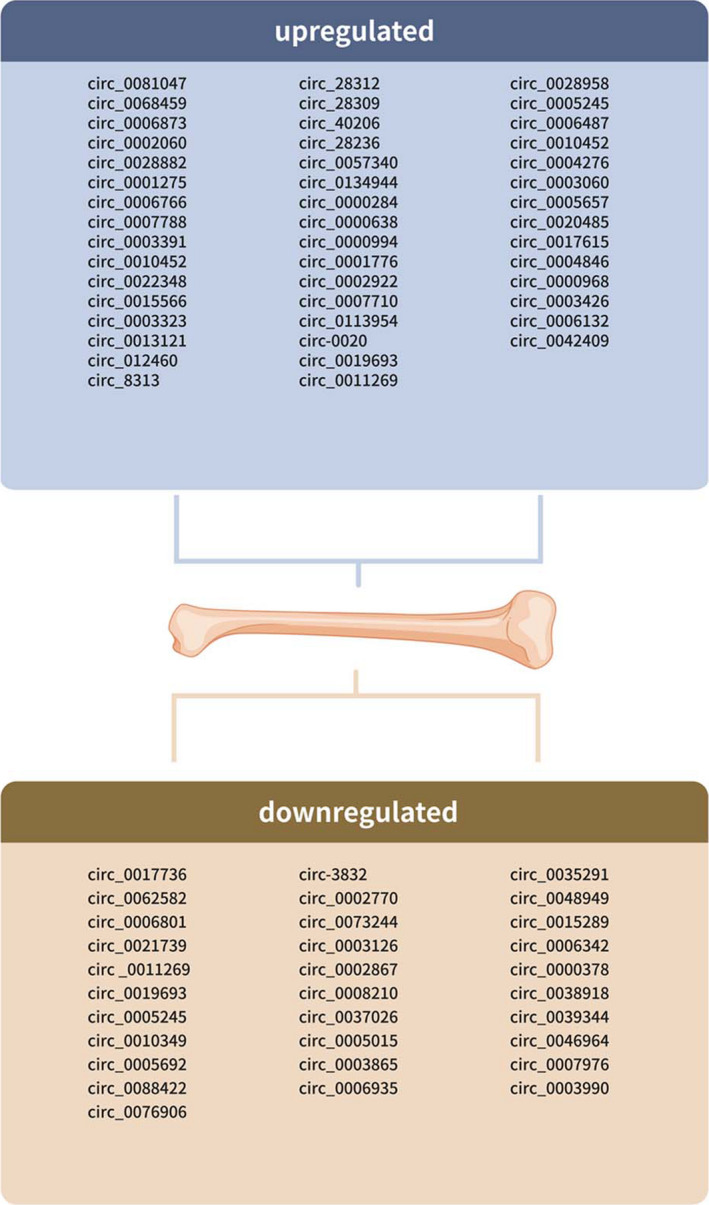
Dysregulated circRNAs in osteoporosis. Until now, there are 34 circRNAs were upregulated and 31 circRNAs were downregulated in osteoporosis

Zhang et al.^58^ used the circRNA microarray to determine circRNA expression profiles in peripheral blood from osteoporosis patients and healthy controls. A total of 398 circRNAs were differentially expressed in peripheral blood from osteoporosis patients compared to healthy controls. Among these, 203 circRNAs were decreased while 195 circRNAs were overexpressed. Top ten upregulated circRNAs were circ_0004276, circ_0003060, circ_0005657, circ_0020485, circ_0017615, circ_0004846, circ_0000968, circ_0003426, circ_0006132 and circ_0042409 and ten downregulated circRNAs were circ_0035291, circ_0048949, circ_0015289, circ_0006342, circ_0000378, circ_0038918, circ_0039344, circ_0046964, circ_0007976 and circ_0003990 (Tables 1 and 2, Figures 1 and 2).

**TABLE 1:** circRNAs expression profiles in osteoporosis

Num	Method	Sample	Upregulated	Downregulated	Reference
1	Microarray RT‐PCR	Plasma	162 circRNAs	95 circRNAs	45
2	Microarray RT‐PCR	PBMCs	203 circRNAs	178 circRNAs	46
3	RNA‐sequencing RT‐PCR	Plasma	106 circRNAs	154 circRNAs	47
4	Microarray RT‐PCR	Bone marrow monocyte	29 circRNAs	52 circRNAs	48
5	RNA‐sequencing RT‐PCR	Plasma	211 circRNAs	176 circRNAs	49
6	Microarray RT‐PCR	OPCSs	95 circRNAs	15 circRNAs	50
7	RNA‐sequencing RT‐PCR	BMSCs	24 circRNAs	21 circRNAs	51
8	Microarray RT‐PCR	Plasma	34 circRNAs	35 circRNAs	52
9	Microarray RT‐PCR	Bone tissues	2645 circRNAs	2327 circRNAs	53
10	Microarray RT‐PCR	Plasma	554 circRNAs	330 circRNAs	54
11	RNA‐sequencing RT‐PCR	Plasma	186 circRNAs	64 circRNAs	55
12	RNA‐sequencing RT‐PCR	BMSCs	173 circRNAs	36 circRNAs	56
13	Microarray RT‐PCR	Serum	376 circRNAs	213 circRNAs	57
14	Microarray RT‐PCR	Peripheral blood	195 circRNAs	203 circRNAs	58

Abbreviations: OC, osteoclast; OPCSs, precursors.

**TABLE 2:** CircRNAs identified from RNA‐sequencing or microarray were confirmed by RT‐qPCR in osteoporosis

Num	Method	Sample	Upregulated	Downregulated	Reference
1	Microarray RT‐PCR	Plasma	circ_0081047, circ_0068459, circ_0006873 circ_0002060	circ_0017736 circ_0062582	45
2	Microarray RT‐PCR	PBMCs	circ_0028882, circ_0001275, circ_0006766, circ_0007788 circ_0003391	circ_0006801	46
3	RNA‐sequencing RT‐PCR	Plasma	circ_0010452, circ_0022348, circ_0015566, circ_0003323 circ_0013121	circ_0021739, circ _0011269, circ_0019693, circ_0005245 circ_0010349	47
4	Microarray RT‐PCR	Bone marrow monocyte	circ_012460, circ_8313, circ_28312, circ_28309, circ_40206, circ_28236		48
5	RNA‐sequencing RT‐PCR	Plasma	circ_0057340 circ_0134944	circ_0005692, circ_0088422 circ_0076906	49
6	Microarray RT‐PCR	OPCSs	circ_0000284, circ_0000638, circ_0000994, circ_0001776, circ_0002922, circ_0007710, circ_0113954		50
7	RNA‐sequencing RT‐PCR	BMSCs	circ‐0020	circ‐3832	51
8	Microarray RT‐PCR	Plasma	circ_0019693, circ_0011269, circ_0028958, circ_0005245, circ_0006487 circ_0010452		52
9	RNA‐sequencing RT‐PCR	BMSCs		circ_0002770, circ_0073244, circ_0003126, circ_0002867, circ_0008210, circ_0037026, circ_0005015, circ_0003865 circ_0006935	56
10	Microarray RT‐PCR	Peripheral blood	circ_0004276, circ_0003060, circ_0005657, circ_0020485, circ_0017615, circ_0004846, circ_0000968, circ_0003426, circ_0006132 circ_0042409	circ_0035291, circ_0048949, circ_0015289, circ_0006342, circ_0000378, circ_0038918, circ_0039344, circ_0046964, circ_0007976 circ_0003990	58

Abbreviations: OC, osteoclast; OPCSs, precursors.

## 3 MECHANISMS OF ACTION OF FUNCTIONALLY IMPORTANT CIRCRNAS IN SCI

### 3.1 circ_0002060 and circ_0006873

Huang et al.^45^ performed circRNA chip method to study circRNA expression profiling in the plasma or serum from 40 osteoporosis patients and 40 control adults, and their data indicated that circ_0006873 and circ_0002060 were upregulated in osteoporosis patients compared to control adults. The level of circ_0002060 and circ_0006873 wa*s* correlated with bone mineral density (BMD) and T‐score. The circ_0002060 levels have diagnostic values for osteoporosis patients (AUC = 0.746, *p* < 0.05), and the specificity and sensitivity for circ_0002060 were 69% and 78%.

### 3.2 Circ_0001275

Zhao et al.^59^ performed circRNA microarray to analysis circRNA expression profiling in PBMCs from three postmenopausal osteoporosis patients and three controls and identified the circ_0001275 was upregulated in osteoporosis group. The level of circ_0001275 was associated with T‐score, and circ_0001275 level was not correlated with weight, height, WBC, age, monocyte count, β‐CROSSL, BMD, lymphocyte, OSTEOC and TP1NP. Moreover, circ_0001275 level in the PBMCs can distinguish people with postmenopausal osteoporosis from controls (AUC: 0.759, *p* < 0.01). These data suggested that circ_0001275 may be one diagnostic biomarker for postmenopausal osteoporosis.

### 3.3 circ_28313

Chen et al.^48^ showed that circ_28313 was overexpressed in the BMM cells after induction with CSF1 and RANKL. Knock‐down of circ_28313 suppressed CSF1 + RANKL‐induced osteoclasts differentiation within the BMM cells in vitro, while inhibited ovariectomized‐influenced (OVX) bone resorption in vivo in mice model. By bioinformatics analysis, it is showed that miR‐195a may bind to CSF1 and circ_28313 and form one miRNA‐circRNA‐mRNA network. circ_28313 relieves miR‐195a‐regulated inhibition on the CSF1 through sponging as one ceRNA, then regulating the osteoclast differentiation in the BMM cells. In summary, circ_28313, CSF1 and miR‐195a act as a ceRNA network in CSF1+ RANKL‐induced osteoclast differentiation, therefore influencing OVX‐induced bone absorption.

### 3.4 circ_0016624

Yu et al.^60^ demonstrated that circ_0016624 was downregulated in the osteoporotic patient samples compared to healthy controls. They indicated that miR‐98 may be one target of circ_0016624 and miR‐98 was upregulated in osteoporosis group compared to controls. Moreover, they found that BMP2 was decreased in osteoporosis group compared to controls. The expression of BMP2 and circ_0016624 was overexpressed while miR‐98 level was downregulated during osteogenic differentiation. Ectopic expression of circ_0016624 can suppress miR‐98 expression and enhance BMP2 expression. Furthermore, circ_0016624 overexpression induced osteogenesis differentiation but decreased through miR‐98 mimics. These data suggested that circ_0016624 inhibited osteoporosis through sponging miR‐98 and promoting BMP2 expression.

### 3.5 circ_0006393

Wang et al.^61^ demonstrated that circ_0006393 was decreased in glucocorticoid‐induced osteoporosis (GIOP) patients compared to presenting traumatic fractures patients. Overexpression of circ_0006393 induced osteogenic genes including BMP2, OPG, Sp7 and RUN X2 expression involved in the bone remodelling. Furthermore, circ_0006393 was found to be localized in the nucleus and cytoplasm of BMSCs. In addition, they found that miR‐145‐5p was a direct target of circ_0006393. Ectopic expression of circ_0006393 induced osteogenic genes including BMP2, OPG, Sp7 and RUN X2 expression via sponging miR‐145‐5p and enhancing FOXO1 expression.

### 3.6 circ_0076906

Wen et al.^62^ found that circ_0076906 was downregulated in the serum and bone tissue of osteoporosis patients. The level of circ_0076906 was upregulated in MSCs after induced via osteogenic, which indicated that circ_0076906 is one negative modulator of osteoporosis. Knock‐down of circ_0076906 inhibited osteoblast differentiation genes including OCN and RUNX2 as well as Alizarin red staining and ALP activity. These data suggested that circ_0076906 inhibited MSCs differentiation to osteogenic. Moreover, miR‐1305 was direct target of circ_0076906 and miR‐1305 was upregulated expression in osteoporosis cases compared to non‐osteoporosis controls. In addition, they showed that circ_0076906 sponged miR‐1305 and inhibited its function, and functioned as one sponge to modulate MSCs differentiation through regulating miR‐1305/OGN expression. In conclusion, they demonstrated that circ_0076906 reduced osteoporosis and induced osteogenic differentiation via regulating miR‐1305/OGN axis.

### 3.7 circ_0048211

Qiao et al.^63^ demonstrated that BMP2 and circ_0048211 were decreased, while miR‐93‐5p was overexpressed in BMSCs isolated from postmenopausal osteoporosis patients. The expression of miR‐93‐5p, BMP2 and circ_0048211 was time‐dependently changed varieties in the BMSCs undergoing osteogenesis. Ectopic expression of circ_0048211 induced OPN, RUNX2 and OCN expression, which also induced mineralization ability and ALP activity. Moreover, they found that circ_0048211 can sponge miR‐93‐5p expression and BMP2 was one direct target gene of miR‐93‐5p. The expression of circ_0048211 was positively associated with BMP2 and negatively associated with miR‐93‐5p. Besides, circ_0048211/miR‐93‐5p/BMP2 regulatory loop was responsible for modulating OPN, RUNX2 and OCN expression and mineralization ability and ALP activity in BMSCs. In summary, their data suggested that circ_0048211 alleviating postmenopausal osteoporosis progression via regulating miR‐93‐5p/BMP2 axis.

### 3.8 circ‐Rtn4

Cao et al.^64^ showed that TNF‐α dose‐dependently promoted the expression of miR‐146a, suppressed cell proliferation, and induced cell apoptosis, as noted via increased Bax protein, cleaved caspase‐3 and caspase‐3 and caspase‐3 activity. Silencing of miR‐146a co‐culture with the BMSCs‐Exos attenuated these functions. Furthermore, co‐culture with the circ‐Rtn4‐treated BMSCs decreased TNF‐α‐induced apoptosis and cytotoxicity in the MC3T3‐E1 cell and it can suppress cleaved caspase‐3, Bax protein expression and caspase‐3 expression and activity. Moreover, miR‐146a was one target gene of circ‐Rtn4, and Rtn4‐Exos displayed its function in the TNF‐α‐induced MC3T3‐E1 cells through sponging miR‐146a. Their data indicated that Rtn4‐Exos attenuated the TNF‐α‐ influenced apoptosis and cytotoxicity in MC3T3‐E1 cells via regulating miR‐146a.

### 3.9 circ_0011269

Xu et al.^52^ demonstrated that circ_0011269 expression was downregulated in the osteoporosis group compared to control group. They found that miR‐122 was one target gene of circ_0011269 and RUNX2 was a target gene of miR‐122. The level of miR‐122 was downregulated during the osteogenic differentiation, while RUNX2 and circ_0011269 level was upregulated. Ectopic expression of circ_0011269 induced RUNX2 expression and suppressed osteoporosis. Above all, their data indicated that circ_0011269 regulated miR‐122/RUNX2 expression and induced osteoporosis progression.

### 3.10 circ_0026827

Ji et al.^56^ showed that circ_0026827 was upregulated in the dental pulp stem cells (DPSCs) during osteoblast differentiation and downregulation expression of circ_0026827 inhibited osteoblast differentiation of DPSCs. Knock‐down of circ_0026827 induced miR‐188‐3p expression and downregulation of miR‐188‐3p restored osteogenic differentiation in DPSCs after treated with circ_0026827 siRNA. Furthermore, they showed that miR‐188‐3p was one target of circ_0026827 and RUNX1 and Beclin1 were target genes of miR‐188‐3p. Overexpression of miR‐188‐3p inhibited osteogenic differentiation of DPSCs through regulating RUNX1 and Beclin1. In addition, they demonstrated that overexpression of circ_0026827 promoted heterotopic bone formation in vivo. Their data suggested that circ_0026827 induced DPSCs differentiation to osteoblast through RUNX1 and Beclin1 signalling pathways via sponging miR‐188‐3p.

### 3.11 circ_0076690

Han et al.^53^ demonstrated that circ_0076690 level was downregulated in the osteoporosis group compared to control group. The level of circ_0076690 was significantly associated with T‐score and BMD, while circ_0076690 expression was not correlated with age and BMI. The AUC value for circ_0076690 was 0.8299 with 85% specificity and 79% sensitivity. They showed that circ_0076690 played one sponge for miR‐152. The level of miR‐152 was downregulated, and circ_0076690 expression was upregulated during osteogenic differentiation. Overexpression of circ_0076690 induced osteogenic differentiation through sponging miR‐152.

### 3.12 circ_0024097

Huang et al.^65^ showed that circRNA YAP1 level was upregulated in MC3T3‐E1 and BMSC during differentiation. Overexpression of YAP1 promoted ALP activity and staining and Runx2 OPN and OCN expression, and it indicated that YAP1 induced osteogenic differentiation of MC3T3‐E1 and BMSCs. circ_0024097 come of YAP1 regulated miR‐376b‐3p to induce YAP1 expression in MC3T3‐E1 and BMSCs. Moreover, YAP1 regulated circ_0024097‐induced osteogenic differentiation and circ_0024097 promoted osteogenic differentiation through activating Wnt/β‐catenin signal pathway. Their data suggested that circ_0024097 suppressed osteoporosis via inducing osteogenic differentiation through Wnt/β‐catenin pathway and miR‐376b‐3p/YAP1 axis.

### 3.13 Circ‐SLC8A1

Lin et al.^66^ demonstrated that circ‐SLC8A1 level was upregulated in the ovariectomy group compared to control group, and the expression of circ‐SLC8A1 was overexpressed in the BMSCs derived from ovariectomy group compared to control group, and circ‐SLC8A1 was upregulated in BMSCs after treated with osteogenic induced medium. Ectopic expression of circ‐SLC8A1 promoted ALP, BGLAP, SPP1 and BMP4 expression and silenced circ‐SLC8A1 inhibited ALP, BGLAP, SPP1 and BMP4 expression, and it suggested that circ‐SLC8A1 acted as promotive role in the development of osteoporosis. They showed that circ‐SLC8A1 sponged miR‐516b‐5p in BMSCs, and the expression of miR‐516b‐5p was downregulated in the BMSCs from ovariectomy group. Circ‐SLC8A1 acted as a promotive role in the development of osteoporosis through regulating miR‐516b‐5p/AKAP2.

### 3.14 CircFOXP1

Shen et al.^44^ showed that circFOXP1 was decreased in osteoporosis bone tissues compared to control group and circFOXP1 acted as a sponge to regulated miR‐33a‐5p expression and induced its target gene FOXP1 expression. The level of FOXP1 and circFOXP1 was upregulated in MSCs during osteogenic differentiation, whereas the expression of miR‐33a‐5p was downregulated. Ectopic expression of circFOXP1 induced FOXP1 expression and inhibited miR‐33a‐5p expression. Overexpression of circFOXP1 induced MSCs differentiated to osteogenic through miR‐33a‐5p/FOXP1 axis in vitro and in vivo. Thus, it suggested that circFOXP1 prevent osteoporosis development and can act as potential therapeutic target for osteoporosis.

### 3.15 Circ_0001445

Xiang et al.^67^ demonstrated that the circ_0001445 level in plasma was downregulated in postmenopausal osteoporosis patients compared to healthy controls and osteopenia patients. The expression of circ_0001445 was positively associated with T‐score and was negatively associated with β‐CTX. It also can distinguish osteopenia patients or/and postmenopausal osteoporosis patients from healthy controls. Furthermore, the circ_0001445 expression was overexpressed in the plasma of postmenopausal osteoporosis patients after treated with anti‐osteoporotic. The level of circ_0001445 was overexpressed in the postmenopausal osteoporosis patients' plasma after the anti‐osteoporotic treatment. It suggested that circ_0001445 in the plasma can act as one new diagnostic biomarker for postmenopausal osteoporosis.

### 3.16 circ_0007059

Liu et al.^55^ found that circ_0007059 was overexpressed in postmenopausal osteoporosis patients and in BMSCs during osteoclastogenesis. Overexpression of circ_0007059 decreased BMSC differentiation into the osteoclasts. Ectopic expression of circ_0007059 promoted TRAP staining and osteoclast‐specific genes expression and suppressed BMP‐2 expression. They also demonstrated that circ_0007059 sponged miR‐378 expression and then targeted BMP‐2 expression. Their data suggested that circ_0007059 acted crucial roles in the osteoclastogenesis through miR‐378/BMP‐2 axis.

### 3.17 CircHmbox1

Liu et al.^68^ demonstrated that circHmbox1 level was decreased in the TNF‐a‐treated osteoclast formation in vitro and in vivo. CircHmbox1 suppressed RANKL‐induced BMMs osteoblasts differentiation partly via binding to miR‐1247‐5p. TNF‐a inhibited osteoblasts differentiation through exosome with the low expression of circHmbox1 from osteoclasts. Moreover, miR‐1247‐5p modulated osteoblasts and osteoclasts differentiation via regulating Bcl6, which was proved to act an opposite role in osteoclasts differentiation and osteoblasts differentiation. Their data suggested that circHmbox1‐sponging miR‐1247‐5p was involved in the modulation of bone metabolisms via TNF‐a in postmenopausal osteoporosis.

### 3.18 circ_0003865

Wang et al.^69^ demonstrated that the circ_0003865 expression was downregulated in the BMSCs after treated with melatonin. Knock‐down of circ_0003865 promoted the expression of OPN, ALP and RUNX2 in BMSCs. Their data suggested that circ_0003865 played as one negative modulator of BMSCs osteogenic differentiation. Melatonin induced BMSC osteogenic differentiation through inhibiting circ_0003865 expression. Circ_0003865 sponges miR‐3653‐3p to modulate GAS1 expression and BMSC osteogenic differentiation. They demonstrated that melatonin promoted BMSCs osteogenic differentiation an inhibited osteoporosis progression via suppressing circ_0003865 expression, which sponges miR‐3653‐3p to promote GAS1 expression and inhibit osteogenic marker genes expression.

### 3.19 circ_0006859

Zhi et al.^70^ demonstrated that circ_0006859 was overexpressed in the exosomes from serum of osteoporosis patients compared to healthy controls. circ_0006859 differentiated osteoporosis or osteopenia cases with high specificity and sensitivity. Ectopic expression of circ_0006859 induced audiogenic differentiation and inhibited osteoblastic differentiation of BMSCs. Moreover, circ_0006859 sponged miR‐431‐5p expression and ROCK1 was one target gene of miR‐431‐5p. Furthermore, they found that circ_0006859 induced adipogenesis and inhibited osteogenesis through regulating miR‐431‐5p to induce ROCK1 expression. These data suggested that circ_0006859 is one biomarker for the postmenopausal osteoporosis and modulated the balance between adipogenesis and osteogenesis in BMSCs through regulating miR‐431‐5p/ROCK1.

### 3.20 circ_0006215

Ji et al.^49^ demonstrated that circ_0006215 expression was downregulated in the BMSCs from cases of senile osteoporosis compared to controls. Ectopic expression of circ_0006215 induced the BMSCs differentiation to osteogenic. RNA pull‐down and Luciferase reporter assays demonstrated that circ_0006215 sponged miR‐942‐5p and then regulated VEGF and RUNX2 expression in the BMSCs. Moreover, they showed that circ_0006215 induced bone defect repair in vivo. These data suggested that circ_0006215 act a critical role in the osteogenesis and may be one therapy target for senile osteoporosis.

### 3.21 circ_0021739

Guan et al.^71^ demonstrated that circ_0021739 was downregulated in the postmenopausal osteoporosis patients compared to controls. The expression of circ_0021739 was associated with femur, forearm and vertebra T‐scores. The AUC of ability of circ_0021739 expression was 0.849, with a specificity of 42.9% and sensitivity of 100%. Ectopic expression of circ_0021739 suppressed miR‐502‐5p expression and suppressed the osteoclasts differentiation. In summary, they demonstrated that circ_0021739 may be one potential biomarker for postmenopausal osteoporosis and circ_0021739 modulated osteoclasts differentiation through regulating miR‐502‐5p.

### 3.22 circ_0001052

Liu et al.^72^ demonstrated that the level of circ_0001052 was downregulated in the BMSCs after treated with low‐level laser irradiation (LLLI). circ_0001052 played as one miR‐124‐3p sponge and suppressed miR‐124‐3p expression. circ_0001052 can modulate BMSCs proliferation through playing as one miR‐124‐3p sponge via the Wnt4/β‐catenin pathway.

### 3.23 Circ_0062582

Li et al.^46^ showed that the expression of circ_0062582 was upregulated in BMSCs during osteogenic differentiation. Overexpression of circ_0062582 induced osteogenic differentiation and promoted the expression of osteogenic differentiation‐associated genes such as COL1, OCN and OSX. Furthermore, they found that circ_0062582 sponged the expression of miR‐145 and CBFB was a direct target gene of the miR‐145. Ectopic expression of circ_0062582 regulated BMSCs osteogenic differentiation through modulating miR‐145/CBFB axis.

### 3.24 circ_0001275

**FIGURE 3:**
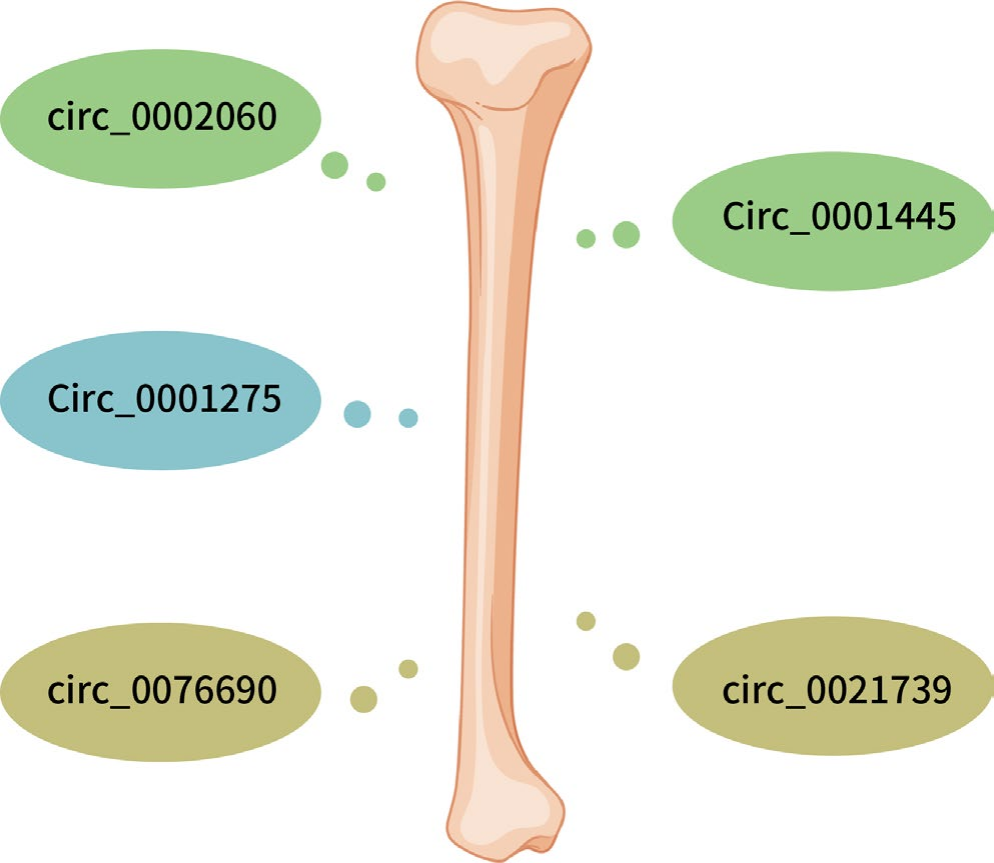
circRNAs acts as biomarkers for osteoporosis. circ_0002060, circ_0001275, circ_0076690, circ_0001445 and circ_0021739 may be one potential biomarkers for osteoporosis

**FIGURE 4:**
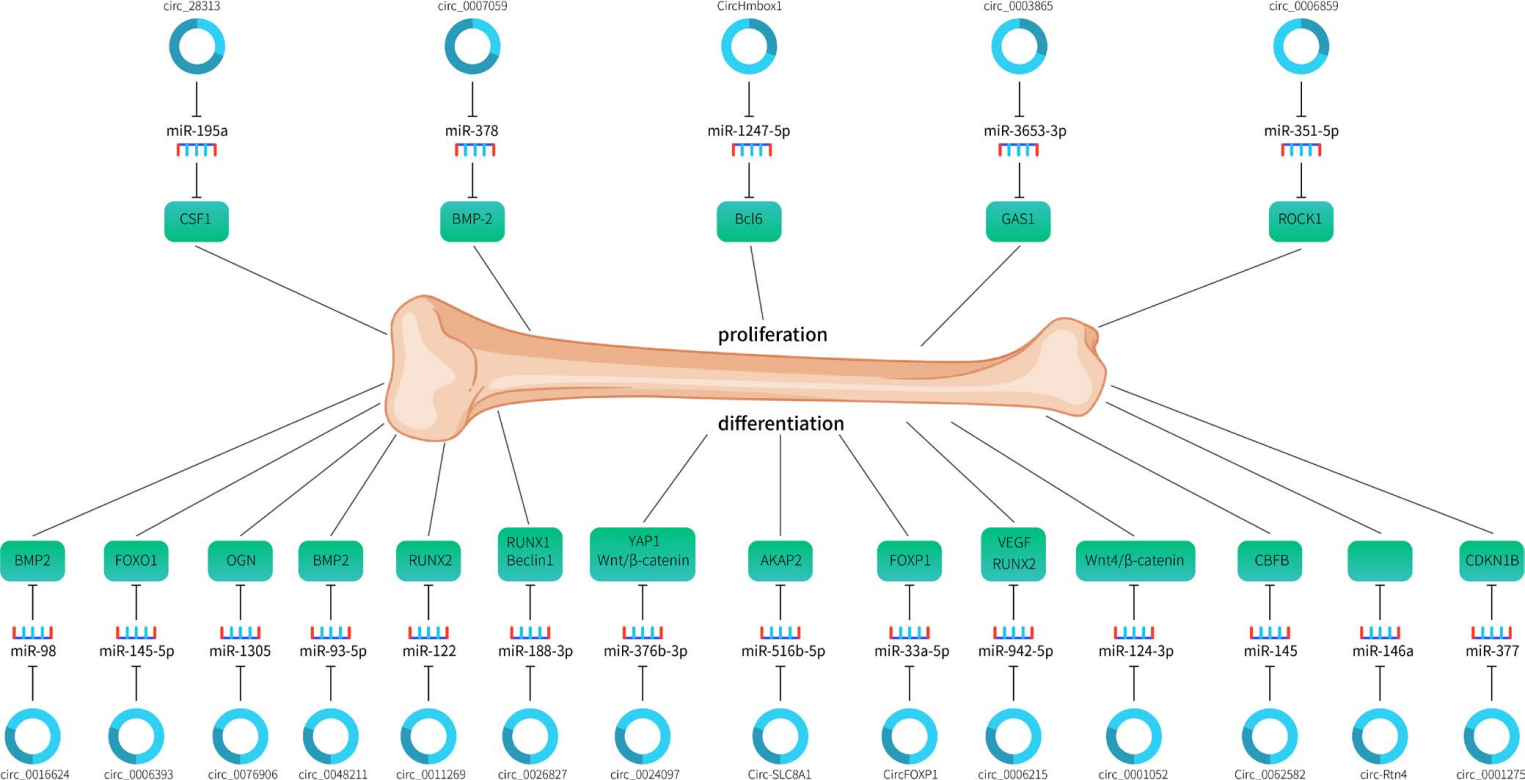
circRNAs regulated genes expression via sponging miRNAs in osteoporosis. Dysregulation expression of circRNA sponged related miRNAs expression and then regulating their target gene involved in the development of osteoporosis. Upper circRNAs represent upregulated in osteoporosis, while below circRNAs represent downregulated

Xu et al.^57^ demonstrated that dexamethasone‐induced the hFOB1.19 cell proliferation inhibition was reversed through silencing circ_0001275. Dexamethasone promoted the calcium nodules and ALP activity in the hFOB1.19 cell, while this function was also reversed by circ_0001275 siRNA. circ_0001275 sponged miR‐377 expression in the hFOB1.19 cells and CDKN1B was one direct target gene of miR‐377. Furthermore, they showed that knock‐down of circ_0001275 reverses dexamethasone‐induced cell proliferation inhibition through promoting miR‐377/CDKN1B axis. Their data suggested that inhibition expression of circ_0001275 can reverse dexamethasone‐induced osteoblast proliferation inhibition through promoting miR‐377/CDKN1B axis (Table 3 and Figures 3 and 4).

**TABLE 3:** Dysregulated circRNAs in osteoporosis

Name	Dysregulation	Sponge target	Function	Related gene	Role	Reference
circ_0002060	Upregulated				Biomarker	45
Circ_0001275	Upregulated				Biomarker	68
circ_28313	Upregulated	miR‐195a	Osteoclast differentiation	CSF1	Harmfulness	48
circ_0016624	Downregulated	miR‐98	Osteogenesis differentiation	BMP2	Protective	60
circ_0006393	Downregulated	miR‐145‐5p	Osteogenesis differentiation	FOXO1	Protective	61
circ_0076906	Downregulated	miR‐1305	MSCs differentiation	OGN	Protective	62
circ_0048211	Downregulated	miR‐93‐5p	MSCs differentiation	BMP2	Protective	63
circ‐Rtn4		miR‐146a	Differentiation		Protective	64
circ_0011269	Downregulated	miR‐122	Differentiation	RUNX2	Protective	52
circ_0026827	Downregulated	miR‐188‐3p	Differentiation	RUNX1 Beclin1	Protective	56
circ_0076690	Downregulated	miR‐152	Differentiation		Protectivebiomarker	53
circ_0024097	Downregulated	miR‐376b‐3p	Differentiation	YAP1 Wnt/β‐catenin	Protective	65
Circ‐SLC8A1	Downregulated	miR‐516b‐5p	Differentiation	AKAP2	Protective	66
CircFOXP1	Downregulated	miR‐33a‐5p	Differentiation	FOXP1	Protective	44
Circ_0001445	Downregulated				Biomarker	67
circ_0007059	Upregulated	miR‐378	Differentiation	BMP‐2	Harmfulness	55
CircHmbox1	Upregulated	miR‐1247‐5p	Differentiation	Bcl6	Harmfulness	68
circ_0003865	Upregulated	miR‐3653‐3p	Differentiation	GAS1	Harmfulness	69
circ_0006859	Upregulated	miR‐431‐5p	Differentiation	ROCK1	Harmfulness	70
circ_0006215	Downregulated	miR‐942‐5p	Differentiation	VEGF RUNX2	Protective	49
circ_0021739	Downregulated	miR‐502‐5p	Differentiation		Protective biomarker	71
circ_0001052	Downregulated	miR‐124‐3p	Proliferation	Wnt4/β‐catenin	Protective	72
Circ_0062582	Downregulated	miR‐145	Differentiation	CBFB	Protective	46
circ_0001275		miR‐377	Proliferation	CDKN1B	Protective	57

## 4 CONCLUSIONS AND FUTURE PERSPECTIVES

Growing data of high‐throughput RNA sequencing and circRNAs microarray studies indicated that circRNAs are differentially expressed in osteoporosis. CircRNAs may be acted as diagnostic biomarkers for osteoporosis. Increasing studies suggested that circRNAs played important roles in the BMSCs proliferation, differentiation and apoptosis. However, the expression levels and function roles of these differentially expressed circRNAs in osteoporosis remain uncharacterized. Further studies may be required to confirm the expression level of these differentially expressed circRNAs in more samples of human osteoporosis and controls. Moreover, further functional works on these differentially expressed circRNAs are needed to build their potential therapeutic or pathogenic significance.

## CONFLICT OF INTEREST

The authors declare that they have no competing interests.

## AUTHOR CONTRIBUTION

**Weichun Chen:** Data curation (equal); Investigation (equal); Visualization (equal); Writing‐original draft (equal); Writing‐review & editing (equal). **Baozhong Zhang:** Conceptualization (equal); Data curation (equal); Investigation (equal); Software (equal); Writing‐original draft (equal); Writing‐review & editing (equal). **Xiao Chang:** Methodology (equal); Visualization (equal); Writing‐original draft (equal); Writing‐review & editing (equal).

## CONSENT TO PARTICIPATE

Not applicable.

## CONSENT TO PUBLISH

Not applicable.

## Data Availability

Research data are not shared.
